# Which patients are not included in the English Cancer Waiting Times monitoring dataset, 2009–2013? Implications for use of the data in research

**DOI:** 10.1038/bjc.2017.452

**Published:** 2018-01-18

**Authors:** C Di Girolamo, S Walters, C Gildea, S Benitez Majano, M P Coleman, B Rachet, M Morris

**Affiliations:** 1 Cancer Survival Group, Faculty of Epidemiology and Population Health, Department of Non-Communicable Disease Epidemiology, London School of Hygiene & Tropical Medicine, Keppel Street, London WC1E 7HT, UK; 2 Department of Medical and Surgical Sciences, Alma Mater Studiorum, University of Bologna, Via Zamboni, 33, 40126 Bologna, Italy; 3 National Cancer Registration and Analysis Service, Public Health England, Vulcan House Steel, 6 Millsands, Sheffield S3 8NU, UK

**Keywords:** waiting times, survival, England, cancer, socio-demographic characteristics, data completeness, bias, monitoring

## Abstract

**Background:**

Cancer waiting time targets are routinely monitored in England, but the Cancer Waiting Times monitoring dataset (CWT) does not include all eligible patients, introducing scope for bias.

**Methods:**

Data from adults diagnosed in England (2009–2013) with colorectal, lung, or ovarian cancer were linked from CWT to cancer registry, mortality, and Hospital Episode Statistics data. We present demographic characteristics and net survival for patients who were and were not included in CWT.

**Results:**

A CWT record was found for 82% of colorectal, 76% of lung, and 77% of ovarian cancer patients. Patients not recorded in CWT were more likely to be in the youngest or oldest age groups, have more comorbidities, have been diagnosed through emergency presentation, have late or missing stage, and have much poorer survival.

**Conclusions:**

Researchers and policy-makers should be aware of the limitations in the completeness and representativeness of CWT, and draw conclusions with appropriate caution.

Successive national cancer plans and strategies for England have included targets to reduce waiting times to diagnosis and treatment for all cancers (Department of Health, 2000, 2007, 2011; Independent Cancer Taskforce, 2015). These include a maximum 2-week wait (TWW) between an urgent referral from a general practitioner (GP) to being seen by a specialist, a maximum 62 days from the GP’s urgent referral to the start of first treatment, and a maximum 31 days from the decision to treat a patient to the start of treatment. A new 28-day target to confirm or exclude a cancer diagnosis has also been proposed (Independent Cancer Taskforce, 2015).

Waiting time targets are considered to be important indicators of the quality of cancer care. National cancer waiting time statistics have been published quarterly by NHS England since 2013−2014 (Cancer Waiting Times Team, 2016) and previously by the Department of Health. Performance varies widely across the country: many Clinical Commissioning Groups fall below current operational standards, and adherence to the 62-day target has been decreasing since 2014 (Cancer Waiting Times Team, 2016).

The English National Cancer Waiting Times monitoring dataset (CWT), is the basis for these official statistics. The data are collected by ‘the provider that is commissioned to deliver the activity’ (Cancer Waiting Times Team, 2015). CWT only contains diagnosis and treatment information on cancer patients who were offered treatment within the NHS, including those who refused treatment or were assigned to active monitoring (Cancer Waiting Times Team, 2015), whichever diagnosis route they came through. However, not all eligible cancer patients are included and the extent of incompleteness is unclear. We examine recent CWT data for three cancers, linked to individual cancer patient data, to compare the characteristics of patients who were and were not included in CWT.

## Materials and methods

### Data sources and study population

All adults (15−99 years) who were diagnosed in England during 2009−2013 with colorectal, non-small cell lung or ovarian cancer were included.

CWT diagnosis and treatment data were linked at individual level with the national cancer registry data (including vital status at 31 December 2014), the Hospital Episode Statistics data, and the Routes to Diagnosis dataset (Elliss-Brookes *et al*, 2012). Demographic information included age, sex, and deprivation quintile. Stage at diagnosis and Charlson Comorbidity Index score (Charlson *et al*, 1987) were derived using algorithms applied to these datasets and audit data, where available (Benitez-Majano *et al*, 2016; Maringe *et al*, 2017).

### Statistical analysis

We examined the sociodemographic and clinical characteristics of cancer patients and estimated net survival at 1 year. We report results by patient age group, deprivation quintile, and tumour stage. Net survival can be interpreted as survival from the cancer, accounting for the mortality from other causes, using life tables of the England general population stratified by age, sex, calendar year, and region (Spika, 2015).

## Results

During 2009−2013, 164 890 colorectal, 171 208 non-small cell lung, and 24 545 ovarian cancer patients were registered in England, of whom 82%, 76%, and 77%, respectively, were included in CWT for first and/or subsequent treatments (Table 1).

**Figure 1:**
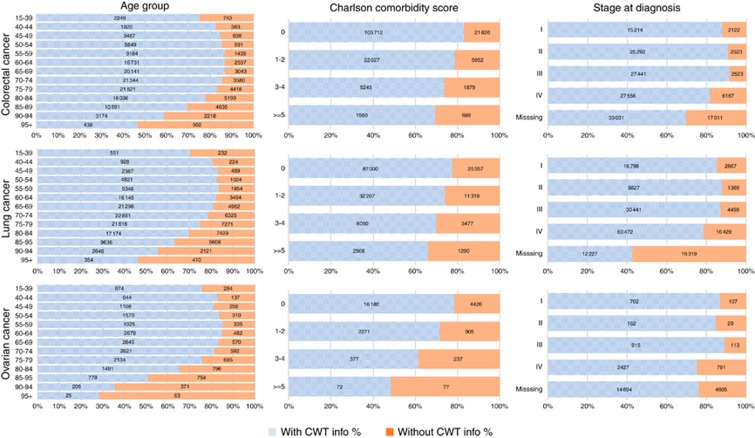
**Age, level of comorbidity, and stage at diagnosis of colorectal, lung, and ovarian cancer patients by presence of a matching record in the Cancer Waiting Times monitoring dataset.**

The completeness of CWT improved slightly during 2009−2011, more for lung and ovarian cancers than for colorectal cancer, then plateaued until 2013. The percentage of patients included varied by patient and tumour factors, with nearly all differences statistically significant in a *χ*^2^-test at *P*<0.001 (Table 1, Figure 1).

There was a strong J-shaped age pattern in the probability of inclusion in CWT (Figure 1): the youngest and, especially, the oldest patients were least likely to be included. Among those older than 70 years, over a quarter with colorectal cancer and around 40% with lung or ovary cancer had no record in CWT. More affluent patients were less likely to have a CWT record. Women with colorectal cancer were slightly less likely to have a CWT record than men (80.2 *vs* 82.7%), but there was no evidence of a difference between the sexes for lung cancer (*P*=0.211).

A CWT record was missing for more than half of patients whose route to diagnosis was unknown, and for around a third of those diagnosed through an emergency presentation.

More than 85% of patients with stage I and II tumours were recorded in CWT (Figure 1). Among colorectal and lung cancer patients, those with missing stage were also the most likely to be missing from CWT, although the proportion was similar to patients diagnosed at stage IV. Among women with ovarian cancer, a similar proportion of cases with stage IV and missing stage tumours were not recorded (24%).

Patients with more comorbidity were less likely to be recorded in CWT (Figure 1). For women with ovarian cancer, those with no comorbidities were 30% more likely to have a CWT record than those with the most comorbidity.

Patients who died within 30 days of diagnosis were much less likely to have a CWT record, ranging from 60% (colorectal and lung cancers) to 73% (ovarian cancer), but 15−18% of those who survived at least 30 days were also not captured (Table 1), with a similar J-shaped age pattern to that of the whole cohort.

For all three cancers, 1-year net survival was far lower among patients who were not captured by CWT than among those who were (colorectal cancer: 52.2% (95% CI, 51.6−52.8%) compared to 81.4% (81.2−81.7%); lung cancer: 17.0% (16.6−17.4%) compared to 39.0% (38.7−39.2%); ovarian cancer: 40.4% (39.1−41.7%) compared to 77.1% (76.4−77.7%); Table 2). The differences generally increased with increasing age, stage, and deprivation. The differences were particularly stark for those with stage IV disease. There was very little difference in survival, among the youngest patients (aged 15−44 years), between those who did and did not appear in CWT.

## Discussion

Using a new approach to examine the English CWT, we linked individual data from several sources to describe the characteristics and short-term survival of patients who were not included in the dataset. Around one-fifth of patients diagnosed with colorectal, lung, or ovarian cancer during 2009–2013 did not have a CWT record. Proportions were highest among elderly patients and those with comorbid conditions, mirroring patterns for those with missing stage information (Adams *et al*, 2004; Worthington *et al*, 2008). Patients missing from CWT were also more likely to have advanced disease or missing stage information: these factors are highly correlated (Barclay *et al*, 2016). However, more than a quarter of the youngest patients and more than 10% of those with early-stage disease also lacked a CWT record, suggesting that the recording of CWT data could be improved.

Several mechanisms may help explain the pattern and extent of missing records. Some treatments may not be well recorded (e.g., pain relief or transfusions). The CWT does not include data on patients who died before treatment could commence, even if a decision to treat had been made. However, in our data, only 22%, 44%, and 31% of colorectal, lung, and ovarian cancer patients without a CWT record, respectively, died within 30 days of diagnosis. Services not commissioned by the NHS are also beyond the scope of CWT data collection, so patients treated in the private sector, including palliative care in non-NHS organisations, are not captured (Cancer Waiting Times Team, 2015). The extent of this is unclear, but it is reported that around 11% of the UK population has some private health insurance (The King's Fund, 2014). The proportion of colorectal and ovarian cancer patients included in CWT was indeed lower among those in the most affluent quintile, who are more likely to seek private care.

A CWT record may be missing because of a clinical decision not to treat a patient: this may explain why older and sicker patients are less likely to be included. Indeed, under- or sub-optimal treatment in the elderly has been reported in England (National Cancer Equality Initiative, 2012; Forrest *et al*, 2014; Lawler *et al*, 2014).

One-year survival was generally much lower among patients with no CWT record, but no survival differences were found among the youngest patients between those with and without a record. This suggests that younger patients may well have had treatment that was not captured in CWT.

There may be some administrative under-reporting by which patients received treatment that was not recorded in CWT. The presence in CWT records of patients who received ‘subsequent treatment’ without any record of first treatment (5–9% depending on the cancer) lends weight to this. The proportion of patients with no CWT record was also higher among those with missing information on stage and with an unknown route to diagnosis, suggesting that there is a group of patients whose information is generally poorly captured, due to shortcomings in data transmission system or clinical documentation.

Our novel approach offers a clear picture of the characteristics of patients who are not included in CWT, but it cannot fully illuminate the mechanisms of this incompleteness. The extent to which the missing data create scope for selection bias must be considered, especially if the reason that patients are not included is related to whether they meet the waiting times target. The generalisability of results from CWT to the whole population of cancer patients recorded in the cancer registration data may be limited, because they do not reflect the outcomes for patients who were treated outside the NHS, or of patients who were not captured despite having received some treatment. These sources of bias should be considered when interpreting the results or using the CWT data to evaluate cancer outcomes at patient level.

The CWT dataset is an important source of data: it allows monitoring of key indicators of NHS performance in cancer care and the targets encourage timely treatment. However, researchers and policy-makers should be aware of these limitations in the completeness and representativeness of the CWT data, and should draw any conclusions with appropriate caution.

## Figures and Tables

**Table 1:** Presence of a record in the CWT by selected characteristics of patients

	**Colorectal cancer**			**Lung cancer**			**Ovarian cancer**		
**Included in CWT dataset**	**Yes,** ***n*** **(%)**	**No,** ***n*** **(%)**	**Total (100%)**	**Yes,** ***n*** **(%)**	**No,** ***n*** **(%)**	**Total (100%)**	**Yes,** ***n*** **(%)**	**No,** ***n*** **(%)**	**Total (100%)**
Total	134 544 (81.6)	30 346 (18.4)	164 890	129 765 (75.8)	41 443 (24.2)	171 208	18 900 (77.0)	5645 (23.0)	24 545
**Year of diagnosis**									
2009	25 731 (79.5)	6616 (20.5)	32 347	23 652 (71.7)	9355 (28.3)	33 007	3630 (72.5)	1374 (27.5)	5004
2010	26 698 (81.8)	5934 (18.2)	32 632	25 047 (74.8)	8421 (25.2)	33 468	3691 (75.3)	1209 (24.7)	4900
2011	27 392 (81.8)	6093 (18.2)	33 485	26 340 (76.6)	8061 (23.4)	34 401	3903 (78.3)	1084 (21.7)	4987
2012	27 945 (82.6)	5880 (17.4)	33 825	27 579 (78.2)	7696 (21.8)	35 275	3850 (79.8)	972 (20.2)	4822
2013	26 778 (82.1)	5823 (17.9)	32 601	27 147 (77.4)	7910 (22.6)	35 057	3826 (79.2)	1006 (20.8)	4832
**Sex**									
Women	58 101 (80.2)	14 369 (19.8)	72 470	58 492 (75.9)	18 535 (24.1)	77 027	18 900 (77.0)	5645 (23.0)	24 545
Men	76 443 (82.7)	15 977 (17.3)	92 420	71 273 (75.7)	22 908 (24.3)	94 181	—	—	—
**Deprivation quintile**									
Least deprived	28 370 (79.9)	7148 (20.1)	35 518	17 880 (74.7)	6060 (25.3)	23 940	3981 (75.9)	1264 (24.1)	5245
2	29 640 (81.7)	6618 (18.3)	36 258	22 338 (75.6)	7221 (24.4)	29 559	4217 (78.6)	1151 (21.4)	5368
3	28 595 (82.2)	6187 (17.8)	34 782	25 788 (75.7)	8256 (24.3)	34 044	3974 (77.2)	1174 (22.8)	5148
4	26 826 (82.5)	5709 (17.5)	32 535	30 690 (75.8)	9788 (24.2)	40 478	3782 (76.9)	1137 (23.1)	4919
Most deprived	21 113 (81.8)	4684 (18.2)	25 797	33 069 (76.6)	10 118 (23.4)	43 187	2946 (76.2)	919 (23.8)	3865
**Route to diagnosis**									
Screening	14 001 (91.4)	1315 (8.6)	15 316	—	—	—	—	—	—
Two-week wait	46 045 (98.5)	722 (1.5)	46 767	45 417 (99.1)	414 (0.9)	45 831	7392 (98.7)	96 (1.3)	7488
Emergency presentation	26 762 (70.3)	11 309 (29.7)	38 071	38 698 (63.8)	21 980 (36.2)	60 678	4879 (65.0)	2630 (35.0)	7509
GP referral	30 583 (77.9)	8667 (22.1)	39 250	25 629 (72.4)	9761 (27.6)	35 390	3859 (72.8)	1439 (27.2)	5298
Inpatient elective	4635 (78.1)	1300 (21.9)	5935	2036 (73.7)	727 (26.3)	2763	195 (69.1)	87 (30.9)	282
Other outpatient^a^	9174 (76.5)	2825 (23.5)	11 999	14 294 (77.8)	4072 (22.2)	18 366	2013 (77.4)	587 (22.6)	2600
Death certificate only^a^	0 (0)	70 (100)	70	2 (1.2)	161 (98.8)	163	0 (0)	15 (100)	15
Unknown	3344 (44.7)	4138 (55.3)	7482	3689 (46.0)	4328 (54.0)	8017	562 (41.5)	791 (58.5)	1353
**Died within 30 days**									
Yes	4307 (39.7)	6543 (60.3)	10 850	13 811 (43.0)	18 326 (57.0)	32 137	635 (26.7)	1741 (73.3)	2376
No	130 237 (84.5)	23 803 (15.5)	154 040	115 954 (83.4)	23 117 (16.6)	139 071	18 265 (82.4)	3904 (17.6)	22 169

Abbreviation: CWT= Cancer Waiting Times monitoring dataset.

All *χ*^2^ *P*-values are *P*<0.001 except deprivation for ovarian cancer: *P*=0.015, and sex for lung cancer: *P*=0.211.

a These categories appear in the Routes to Diagnosis dataset, but are not recorded separately in CWT.

**Table 2:** One-year NS and presence of CWT record by age, stage, and deprivation of patients

	**Colorectal cancer**		**Lung cancer**		**Ovarian cancer**	
	**Yes**	**No**	**Yes**	**No**	**Yes**	**No**
**Included in CWT dataset**	**NS % (95% CI)**	**NS % (95% CI)**	**NS % (95% CI)**	**NS % (95% CI)**	**NS % (95% CI)**	**NS % (95% CI)**
Overall	81.4 (81.2–81.7)	**52.2 (51.6–52.8)**	39.0 (38.7–39.2)	**17.0 (16.6–17.4)**	77.1 (76.4–77.7)	**40.4 (39.1–41.7)**
**Age groups, years**						
15–44	86.9 (85.8–87.9)	87.9 (86.0–89.8)	52.0 (49.4–54.5)	56.6 (52.0–61.1)	90.7 (89.2–92.2)	88.9 (85.9–91.9)
45–54	87.7 (87.0–88.4)	**77.8 (75.8–82.9)**	46.0 (44.9–47.2)	**29.8 (27.5–32.1)**	89.4 (88.2–90.5)	**79.0 (75.6–82.3)**
55–64	87.7 (87.3–88.1)	**71.8 (70.4–73.2)**	44.0 (43.4–44.6)	**22.2 (21.1–23.3)**	84.1 (83.1–85.2)	**59.8 (56.4–63.2)**
65–74	85.2 (84.8–85.6)	**61.2 (60.0–62.4)**	42.1 (41.6–42.6)	**18.1 (17.4–18.8)**	78.0 (76.9–79.2)	**42.8 (39.9–45.7)**
75+	74.0 (73.6–74.4)	**39.2 (38.4–40.0)**	32.4 (31.9–32.8)	**13.6 (13.2–14.1)**	57.3 (55.8–58.8)	**17.6 (16.1–19.1)**
**Stage**						
I	98.4 (98.1–98.8)	**94.0 (92.7–95.3)**	85.2 (84.6–85.8)	**69.9 (68.1–71.7)**	97.4 (96.8–98.1)	95.6 (93.7–97.5)
II	94.6 (94.3–95.0)	**82.7 (81.1–84.4)**	69.0 (68.0–69.9)	**45.1 (42.3–47.8)**	91.0 (89.1–92.9)	**73.9 (66.6–81.1)**
III	89.8 (89.3–90.2)	**71.1 (69.2–73.0)**	44.5 (44.0–45.1)	**19.8 (18.6–21.0)**	80.1 (78.9–81.2)	**47.4 (43.6–51.2)**
IV	52.2 (51.6–52.8)	**21.4 (20.4–22.5)**	19.2 (18.9–19.5)	**7.0 (6.6–7.4)**	62.8 (61.2–64.5)	**20.8 (18.2–23.4)**
Missing	81.0 (80.6–81.4)	**50.8 (50.0–51.5)**	35.1 (34.3–36.0)	**14.7 (14.2–15.3)**	68.2 (67.0–69.5)	**33.4 (31.8–35.1)**
**Deprivation quintile**						
Least deprived	83.5 (83.1–84.0)	**61.5 (60.3–62.7)**	39.9 (39.2–40.6)	**22.2 (21.2–23.3)**	78.7 (77.4–80.1)	**49.4 (46.6–52.2)**
2	82.6 (82.1–83.1)	**55.4 (54.2–56.7)**	39.3 (38.6–39.9)	**18.4 (17.5–19.3)**	76.1 (74.7–77.4)	**44.5 (41.6–47.4)**
3	81.3 (80.8–81.8)	**50.9 (49.6–52.2)**	38.6 (38.0–39.2)	**16.5 (15.7–17.3)**	77.1 (75.7–78.4)	**37.8 (35.0–40.6)**
4	79.9 (79.4–80.5)	**46.3 (44.9–47.7)**	38.4 (37.8–38.9)	**15.9 (15.1–16.6)**	75.9 (74.4–77.3)	**33.9 (31.1–36.8)**
Most deprived	78.9 (78.3–79.5)	**42.2 (40.7–43.7)**	39.0 (38.5–39.6)	**14.5 (13.8–15.2)**	77.8 (76.2–79.4)	**33.9 (30.8–37.1)**

Abbreviations: CI=confidence interval; CWT= Cancer Waiting Times monitoring dataset; NS=net survival.

Bold font indicates that CIs in the ‘No’ column do not overlap with those in the corresponding ‘Yes’ column.
